# Identification of boric acid as a novel chemoattractant and elucidation of its chemoreceptor in *Ralstonia pseudosolanacearum* Ps29

**DOI:** 10.1038/s41598-017-09176-3

**Published:** 2017-08-17

**Authors:** Akiko Hida, Shota Oku, Yutaka Nakashimada, Takahisa Tajima, Junichi Kato

**Affiliations:** Department of Molecular Biotechnology, Graduate School of Advanced Sciences of Matter, Hiroshima University, Higashi-Hiroshima, Hiroshima, 739-8530 Japan

## Abstract

Chemotaxis enables bacteria to move toward more favorable environmental conditions. We observed chemotaxis toward boric acid by *Ralstonia pseudosolanacearum* Ps29. At higher concentrations, the chemotactic response of *R. pseudosolanacearum* toward boric acid was comparable to or higher than that toward L-malate, indicating that boric acid is a strong attractant for *R. pseudosolanacearum*. Chemotaxis assays under different pH conditions suggested that *R. pseudosolanacearum* recognizes B(OH)_3_ (or B(OH_3_) + B(OH)_4_^−^) but not B(OH)_4_^−^ alone. Our previous study revealed that *R. pseudosolanacearum* Ps29 harbors homologs of all 22*R. pseudosolanacearum* GMI1000 *mcp* genes. Screening of 22 *mcp* single-deletion mutants identified the RS_RS17100 homolog as the boric acid chemoreceptor, which was designated McpB. The McpB ligand-binding domain (LBD) was purified in order to characterize its binding to boric acid. Using isothermal titration calorimetry, we demonstrated that boric acid binds directly to the McpB LBD with a *K*_*D*_ (dissociation constant) of 5.4 µM. Analytical ultracentrifugation studies revealed that the McpB LBD is present as a dimer that recognizes one boric acid molecule.

## Introduction

Chemotaxis, a universal phenomenon in motile bacteria, involves sensing of chemical gradients and subsequent navigation toward/away from more favorable/unfavorable environmental conditions^1^. Because most known chemical attractants are growth substrates^2–5^, chemotaxis is believed to enable bacterial cells to efficiently move toward areas that are most suitable for growth. Bacterial chemotaxis also can be viewed as an important initial step in ecological interactions such as symbiosis, root colonization, and infection^6^. Chemotaxis reportedly plays important roles in nodulation involving *Rhizobium leguminosarum* ^7^, root colonization by plant growth–promoting *Pseudomonas fluorescens* ^8–10^, and plant infection by *Ralstonia solanacearum* ^11, 12^.

The molecular mechanisms that underlie bacterial chemotaxis have been studied intensively in *Escherichia coli* and *Salmonella enterica* serovar Typhimurium^13, 14^. Chemotactic ligands are detected by cell surface chemoreceptors known as methyl-accepting chemotaxis proteins (MCPs). Upon ligand binding, MCPs generate chemotaxis signals that are communicated to the flagellar motor via a series of chemotaxis (Che) proteins. In *E. coli*, five MCPs (Tsr, Tar, Trg, Tap, and Aer) and six Che proteins (CheA, CheB, CheR, CheW, CheY, and CheZ) have been identified to date^15^.

*Ralstonia solanacearum* is a gram-negative, motile plant pathogenic bacterium that causes bacterial wilt in a number of economically important crops, including tomato, potato, eggplant, tobacco, and banana^16, 17^. This soil-borne bacterium usually enters plant roots through wounds, the root tips, and secondary root emergence points, eventually invading the xylem vessels and spreading to the aerial parts of the plant^18^. *Ralstonia solanacearum* is a heterogeneous species designated “the *R. solanacearum* species complex”^19, 20^. The *R. solanacearum* species complex is motile and exhibits chemotactic responses to a wide variety of compounds, including amino acids, sugars, dicarboxylic acids, citrate, and inorganic phosphate^11, 12^.

Complete genomic sequences have been determined for several strains of the *R. solanacearum* species complex^21^. These strains express more than 20 MCPs, including two aerotaxis sensors^22^. In a previous study, we demonstrated that *Ralstonia pseudosolanacearum* (formerly *R. solanacearum*) strain Ps29 expresses 22 MCPs, three of which (McpA, McpM, and McpT) have been identified as chemotaxis sensors for amino acids, L-malic acid, and D-malic acid, respectively^12, 23^. Sand-soak virulence assays using single-deletion *mcp* mutants of *R. pseudosolanacearum* strain MAFF106611 revealed that McpM is involved in infection of tomato plants. However, the specific functions and contributions to infection of the other MCPs have yet to be determined.

In the course of our chemotaxis research, we observed a unique phenomenon with *R. pseudosolanacearum* strain Ps29. We used computer-assisted capillary assays to analyze chemotaxis behavior in various bacteria, including *Pseudomonas aeruginosa*, *Pseudomonas putida*, *P. fluorescens*, *and R. pseudosolanacearum*. HEPES buffer was used for cell suspensions and as a negative control for the chemotaxis assays. *Pseudomonas* strains exhibited no response to HEPES buffer, whereas *R. pseudosolanacearum* exhibited weak but significant chemotaxis toward HEPES buffer and occasionally even exhibited strong chemotaxis to the “negative” control. We investigated this phenomenon in detail and found that *R. pseudosolanacearum* was attracted to boric acid. In this study, we describe chemotaxis toward boric acid by *R. pseudosolanacearum* and the identification and characterization of its chemotaxis sensor.

## Results

### Discovery of chemotaxis toward boric acid

**Figure 1:**
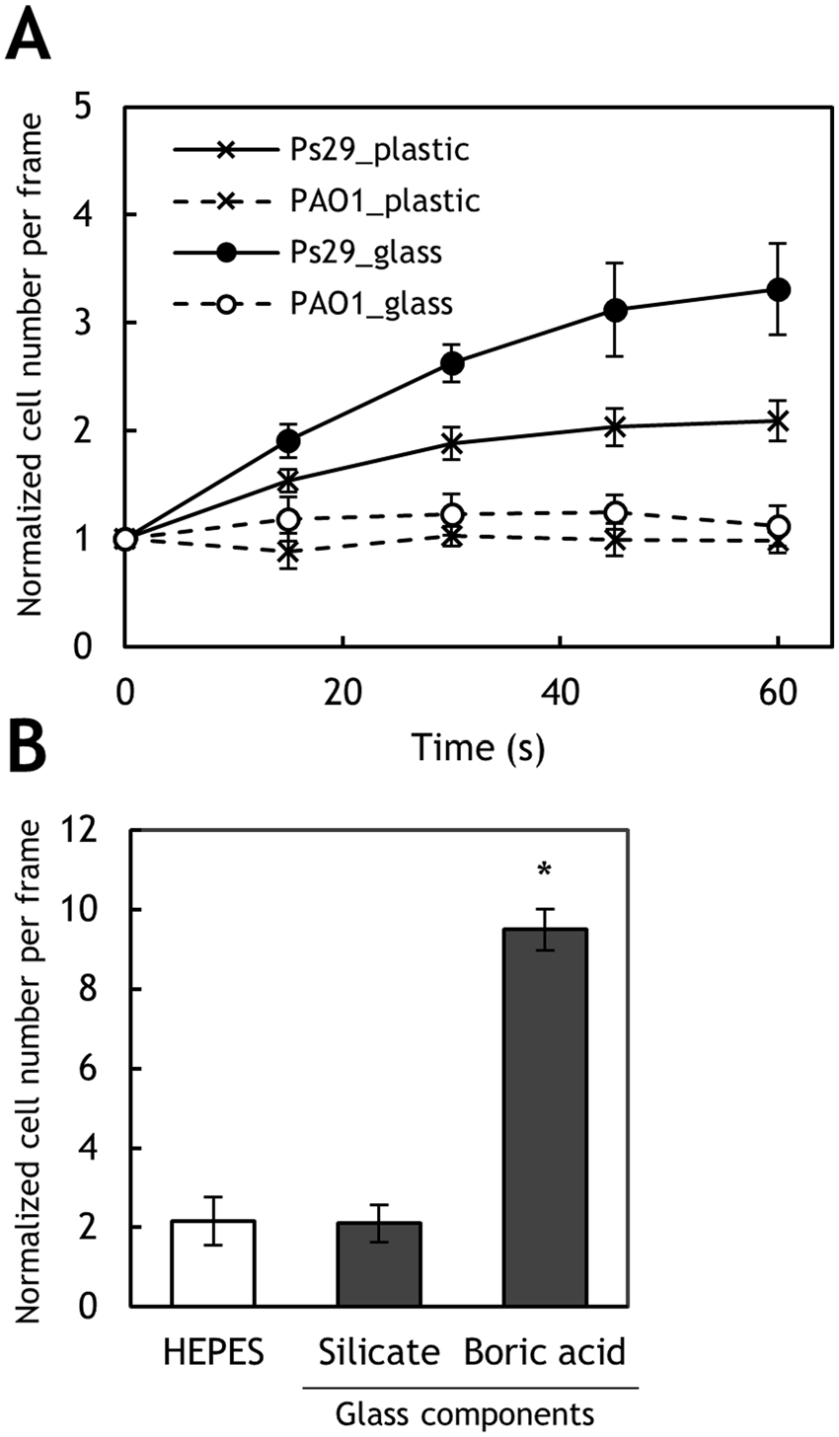
Discovery of chemotaxis to boric acid. (**A**) Chemotaxis toward negative control HEPES buffer by *R. pseudosolanacearum* Ps29 and *P. aeruginosa* PAO1. ‘Plastic’ and ‘glass’ indicate HEPES buffer stored in a plastic tube and glass bottle, respectively. (**B**)  Chemotaxis toward 5 mM silicate and 0.5 mM boric acid by *R. pseudosolanacearum* Ps29. HEPES stored in a plastic tube was used as a control. The normalized cell number was calculated by dividing the number of bacterial cells observed at 1 min by the number observed at the initiation of the experiment. Vertical bars represent the standard error of measurement for experiments performed at least in triplicate. Asterisk indicates a statistically significant difference compared with the response to buffer (*P* < 0.05 by Student’s *t*-test).

We used a computer-assisted capillary assay method^24^ to assess bacterial chemotaxis. In this method, a glass capillary containing a known concentration of a test compound plus 1% agarose in 10 mM HEPES buffer (pH 7.0) is inserted into a bacterial cell suspension. HEPES buffer is also used for cell suspensions. The bacteria sense the test compound diffusing from the orifice of the glass capillary, and if attracted by the test compound, swim toward the orifice of the capillary. HEPES buffer at the same concentration and pH was used as a negative control in the chemotaxis assay. *R. pseudosolanacearum* Ps29 showed a chemotactic response to HEPES buffer although it was weak (Fig. 1A), and sometimes, the bacteria exhibited very strong responses to HEPES buffer. Careful review of the experimental procedure revealed that *R. pseudosolanacearum* exhibited much stronger responses to HEPES buffer stored in a borosilicate glass bottle for an extended time (for example, overnight) than to generally used buffer stored in plastic bottles, and these responses were reproducible. By contrast, *Pseudomonas* strains, including *P. aeruginosa*, *P. fluorescens*, and *P. putida*, exhibited no responses to HEPES buffer stored in either type of container (Fig. 1A). These results suggest that component(s) leaching from the borosilicate glass served as a chemoattractant(s) for *R. pseudosolanacearum* Ps29. Because boric acid and silicate should be the major compounds leaching from borosilicate glass, we assessed the chemotactic response of *R. solanacearum* Ps29 to these compounds. There was no significant difference between the responses to 5 mM silicate and the control (HEPES buffer stored in a plastic tube), but *R. pseudosolanacearum* Ps29 exhibited a significantly stronger response to 0.5 mM boric acid than to HEPES buffer (*P* < 0.05 by Student’s *t* test). This result clearly demonstrates that boric acid is a chemoattractant for *R. pseudosolanacearum* Ps29 and suggests that boric acid leaching from glassware was the cause of the chemotactic response of *R. pseudosolanacearum* Ps29 to the “negative” control. Therefore, all subsequent experiments were carried out without glassware (excepting glass capillaries, cover slips, and the slide glass in the chemotaxis assay).

### Characterization of chemotaxis to boric acid

**Figure 2:**
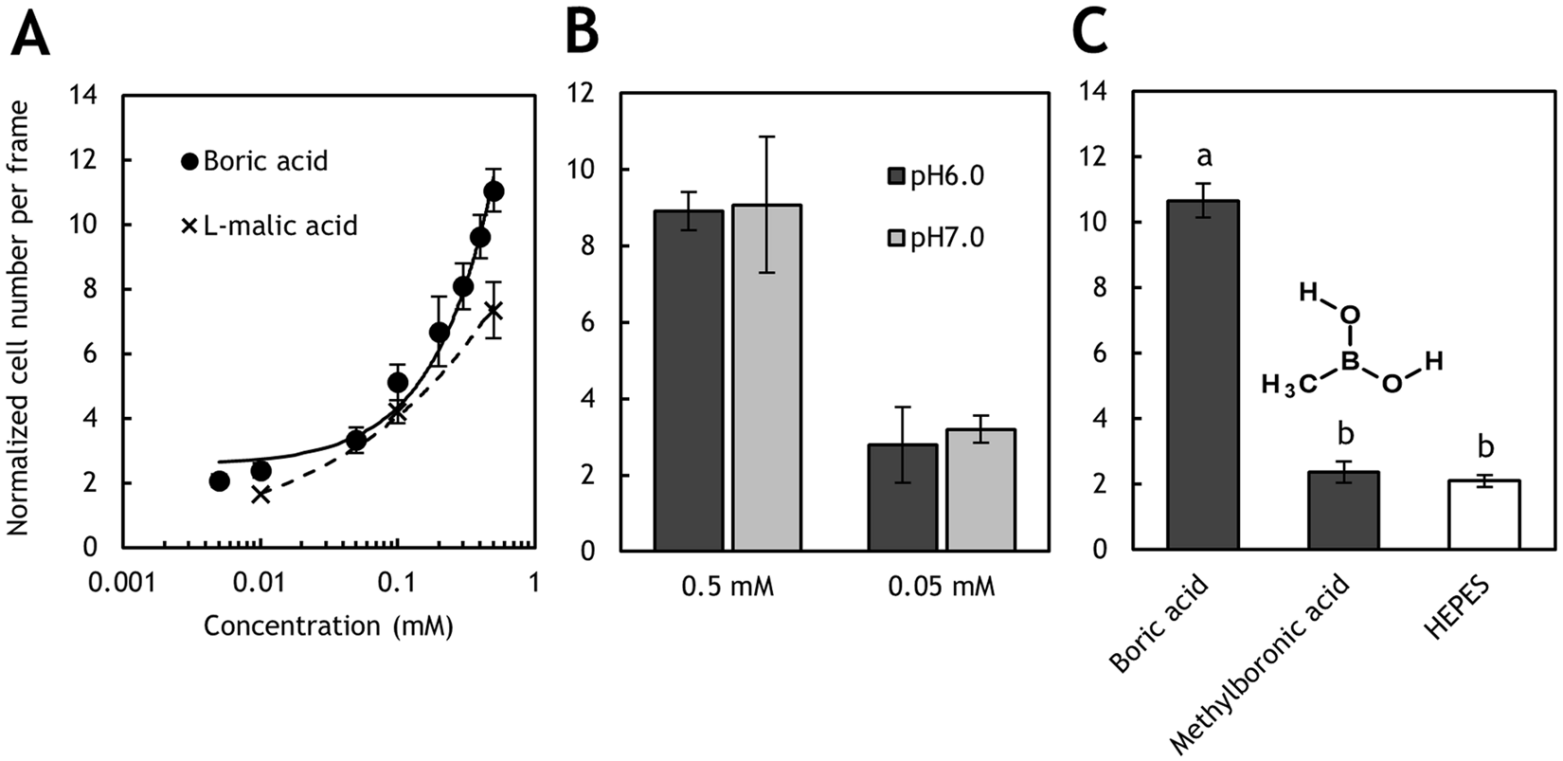
Characterization of chemotaxis toward boric acid by *R. pseudosolanacearum* strain Ps29. (**A**) Concentration-dependent chemotaxis toward boric acid and L-malic acid. (**B**) Chemotaxis toward 0.5 mM boric acid at pH 6.0 and 7.0. (**C**) Chemotaxis toward 0.5 mM boric acid and 0.5 mM methylboronic acid. The normalized cell number was calculated by dividing the number of bacterial cells observed at 1 min by the number observed at the initiation of the experiment. Vertical bars represent the standard error of measurement for experiments performed at least in triplicate. Different letters indicate significant differences (*P* < 0.05 by Student’s *t*-test).

As shown in Fig. 2A, *R. pseudosolanacearum* Ps29 exhibited a concentration-dependent chemotactic response to boric acid. The threshold concentration of boric acid for chemotaxis was 0.01 mM. We previously demonstrated that L-malate strongly attracts *R. pseudosolanacearum* Ps29^12^. In the present study, we found that at high concentrations (>0.1 mM), boric acid elicited a chemotactic response comparable to or stronger than that elicited by L-malate (Fig. 2A), indicating that boric acid is also a strong chemoattractant for *R. pseudosolanacearum* Ps29.

The effect of pH on boric acid chemotaxis is shown in Fig. 2B. There was no significant difference between the strength of chemotaxis at pH 6.0 and 7.0. As a Lewis acid, boric acid abstracts OH from water^25^:

1 $${\rm{B}}{({\rm{OH}})}_{3}+{{\rm{H}}}_{2}{\rm{O}}\leftrightarrow {\rm{B}}{{({\rm{OH}})}_{4}}^{-}+{{\rm{H}}}^{+}({{\rm{pK}}}_{{\rm{a}}}=9.14)$$

The corresponding Henderson-Hasselbalch equation is:

2 $${\rm{pH}}={{\rm{pK}}}_{{\rm{a}}}+\,\mathrm{log}\,([{\rm{B}}{{({\rm{OH}})}_{4}}^{-}]/[{\rm{B}}{({\rm{OH}})}_{3}])$$

Solving eq. 2 indicates that at pH 6.0, a concentration of borate (B(OH)_4_^−^) (0.058% boron) is 10-fold less than at pH 7.0 (0.57% boron). However, the strength of boric acid chemotaxis at pH 6.0 was comparable to that at pH 7.0, which suggests that the chemotaxis sensor detects boric acid (B(OH)_3_) (alternatively, B(OH)_3_ + B(OH)_4_^−^) but not B(OH)_4_^−^ alone.

### Identification of boric acid MCPs

**Figure 3:**
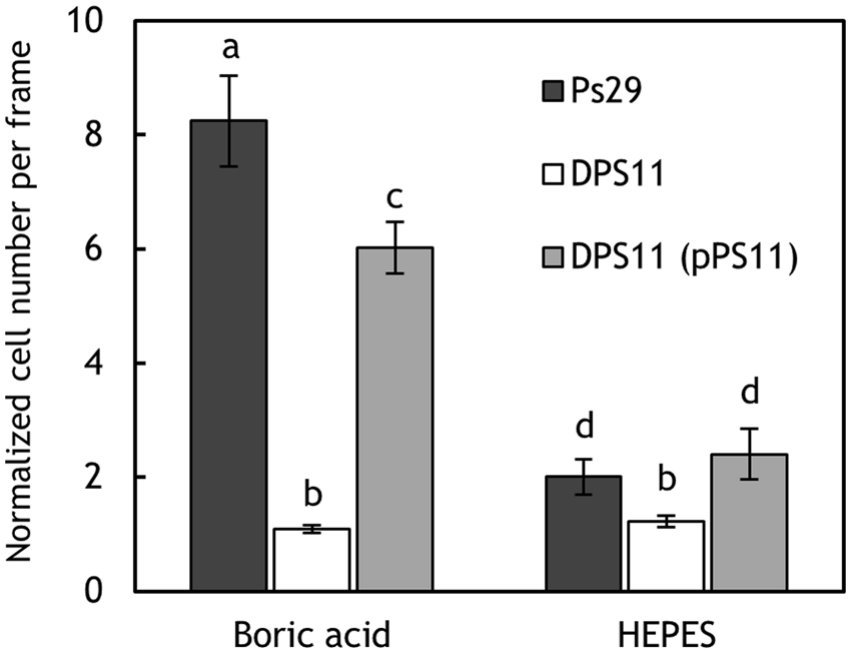
Chemotactic response of *R. pseudosolanacearum* Ps29 strains to 0.1 mM boric acid. Ps29, wild type; DPS11, *mcpB* deletion mutant; DPS11 (pPS11), DPS11 harboring pPS11. The normalized cell number was calculated by dividing the number of bacterial cells observed at 1.5 min by the number observed at the initiation of the experiment. Vertical bars represent the standard error of measurement for experiments performed at least in triplicate. Different letters indicate significant differences (*P* < 0.05 by Student’s *t*-test).

MCPs are transmembrane chemoreceptors that serve as sensor molecules in bacterial chemotaxis. The genome sequence of *R. pseudosolanacearum* (formerly *R. solanacearum*) GMI1000 has been determined^26^ and was found to encode 22 putative *mcp* genes, and *R. pseudosolanacearum* Ps29 harbors homologs to all 22 of these genes^12^. We constructed a library of 22*R. pseudosolanacearum* Ps29 *mcp* single-deletion mutants^12^. To identify the gene encoding the MCP for boric acid, the library of mutants was screened for chemotactic responses to boric acid. Among the *mcp* single-deletion mutants, strain DPS11, in which the RS_RS17100 (old locus tag RSc3412) orthologue was deleted, failed to respond to boric acid (Fig. 3). The introduction of plasmid pPS11, which harbors the RS_RS17100 orthologue of *R. pseudosolanacearum* Ps29, restored the chemotactic response of strain DPS11 to boric acid, demonstrating that the RS_RS17100 homolog encodes an MCP for boric acid. The RS_RS17100 homologous protein of strain Ps29 was 99% identical (512 of 515 amino acids [aa]) to GMI1000 RS_RS17100^12^. We accordingly renamed the RS_RS17100 homolog *‘mcpB’*.

### Ligand specificity of McpB

To investigate ligand specificity of McpB, we measured chemotactic responses of *R. pseudosolanacearum* Ps29 to compounds having similar structures to boric acid. They included methylboronic acid (CH_3_B(OH)_2_), methanediol (CH_2_(OH)_2_) (provided as formaldehyde), methanol (CH_2_OH), aluminum hydroxide (Al(OH)_3_), phosphate, sulfate, and arsenate (both methanetetrol (C(OH)_4_) and methanetriol (CH(OH)_3_) are hypothetical compounds and thus unavailable) (Fig. S1). *R. pseudosolanacearum* Ps29 cells showed weak attractive responses to “a negative control” (HEPES buffer) because they responded to a small amount of boric acid diffused from a glass capillary. Although *R. pseudosolanacearum* Ps29 cells showed a strong responses to 0.5 mM boric acid, methylboronic acid, methanediol, methanol, aluminum hydroxide, and sulfate elicited only basal responses in Ps29 cells (i.e. the responses were not significantly different from that to the negative control). Responses to phosphate and arsenate were significantly higher than that to the negative control. To investigate whether McpB senses phosphate and arsenate, we compared responses of the wild-type strain and *mcpB* mutant of *R. pseudosolanacearum* Ps29 (Fig. S1B). The *mcpB* mutant showed decreased responses to phosphate and arsenate compared to those of the wild-type strain. But, the differences of the strength of chemotaxis to phosphate and arsenate between the wild-type strain and the mutant strain were similar to that to the negative control (Fig. S1B), suggesting that decreased responses of the *mcpB* mutant to phosphate and sulfate were due to the lack of boric acid chemotaxis. When taken together, McpB does not sense these compounds with similar chemical structures.

### Direct binding of boric acid to the McpB ligand-binding domain (LBD)

**Figure 4:**
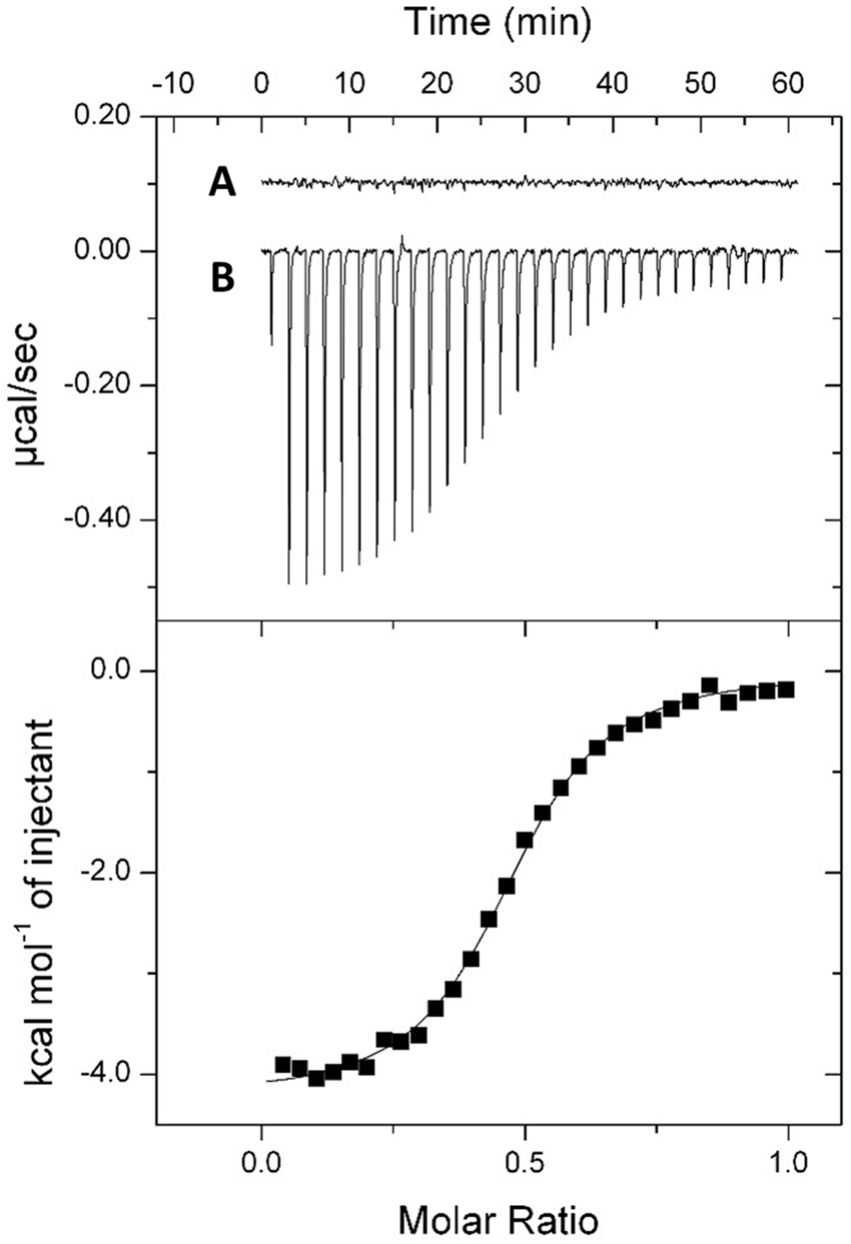
Isothermal titration calorimetry (ITC). A, Titration of ITC buffer with 1 mM boric acid; B, Titration of 200 μM McpB LBD with 1 mM boric acid. Upper panel shows the raw titration data, and lower panel shows the integrated, dilution-corrected and concentration-normalized peak areas of the raw titration data. The data were fitted using the One Set of Sites model of the MicroCal version of Origin7.

McpB shows structural characteristics typical of MCPs: a positively charged N-terminus followed by a hydrophobic membrane-spanning region, a hydrophilic periplasmic domain, a second hydrophobic membrane-spanning region, and a hydrophilic cytoplasmic domain^27^. Chemotactic ligands are known to bind to the periplasmic domains (i.e., LBDs) of MCPs, thereby initiating chemotactic signaling. Dense alignment surface analysis^28^ identified the LBD of McpB as a region spanning 157 aa (residues 33 to 187). To determine whether McpB recognizes boric acid directly, the LBD of McpB was overexpressed and purified from an *Escherichia coli* lysate soluble fraction. Purified McpB LBD was then analyzed using isothermal titration calorimetry (ITC). Titration of buffer with 1 mM boric acid generated almost no heat of dilution (Fig. 4A). By contrast, titration of 200 μM McpB LBD with 1 mM boric acid produced a large heat change that diminished as protein saturation approached (Fig. 4B). Analysis of the data revealed that binding of boric acid to the McpB LBD was driven by a favorable enthalpy change (Δ*H* = −4.52 kcal/mol), with a *K*_*D*_ of 5.44 μM. These results demonstrate that boric acid binds to the LBD of McpB directly.

### Characterization of binding between the McpB LBD and boric acid

**Figure 5:**
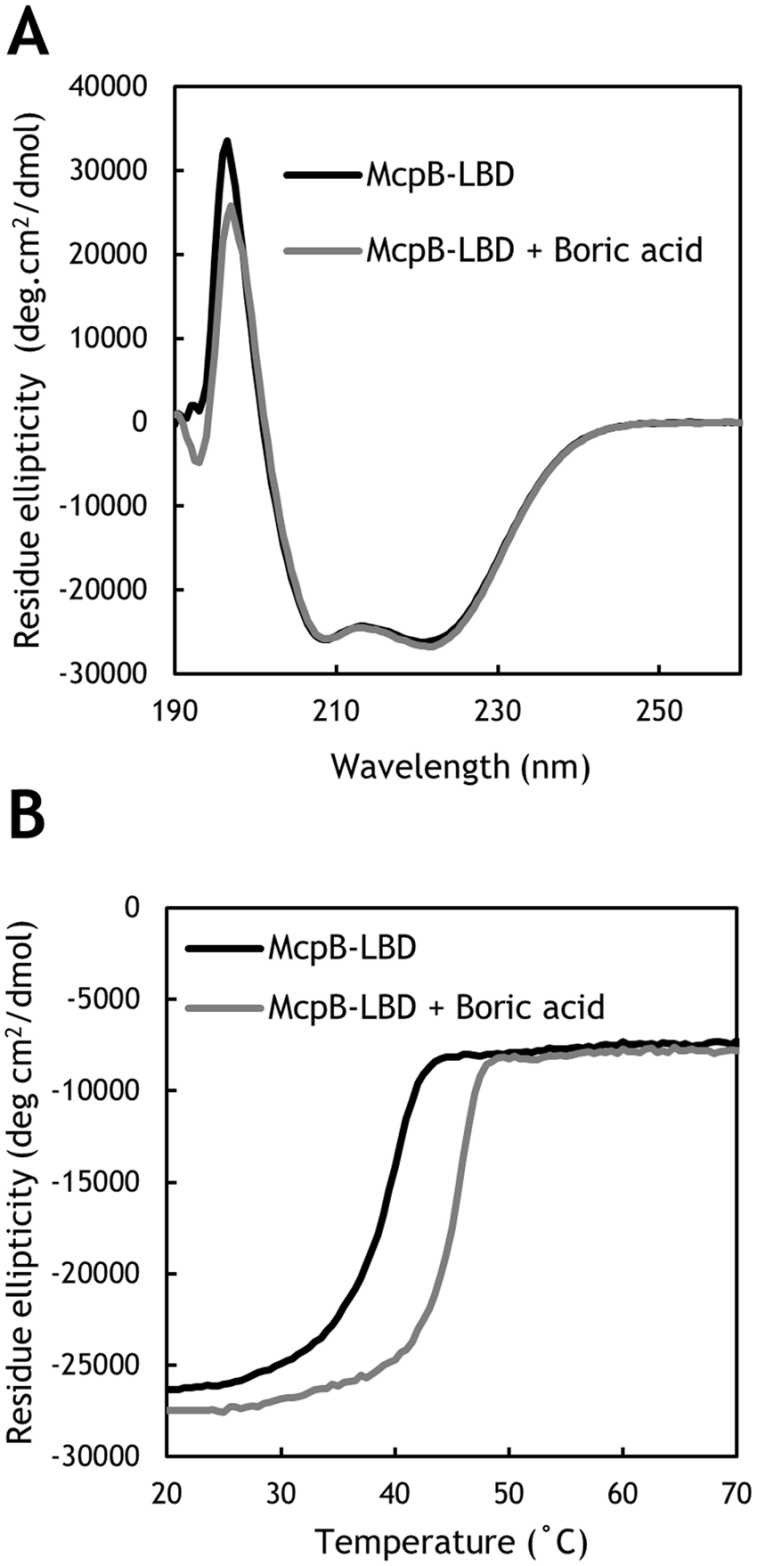
Circular dichroism (CD) spectroscopic analysis of McpB LBD. (**A**) CD spectra of 20 μM McpB LBD in the absence and presence of 100 μM boric acid. (**B**) Thermal denaturation of 20 μM McpB LBD in the absence and presence of 100 μM boric acid as determined by CD ellipticity at 222 nm. Shown are average curves from triplicate experiments.

Several analytical techniques were used to characterize the McpB boric acid–sensing mechanism. The far-UV circular dichroism (CD) spectrum for the McpB LBD showed minima at 208 and 222 nm, which is typical of α-helical proteins (Fig. 5A). The α-helical content of the McpB LBD was calculated at 78%, which was similar to the α-helical content of 89% derived from the McpB LBD model (Fig. S2). The addition of boric acid produced no major changes in the CD spectrum of the McpB LBD, indicating that ligand binding does not significantly alter the McpB LBD secondary structure. Thermal unfolding of the McpB LBD was then assessed by monitoring the CD signal at 222 nm (Fig. 5B). In the absence of boric acid, the midpoint of protein unfolding (T_m_) was 39.3 °C. However, a T_m_ of 45.2 °C was observed in the presence of boric acid, corresponding to an increase of approximately 6 °C.

**Figure 6:**
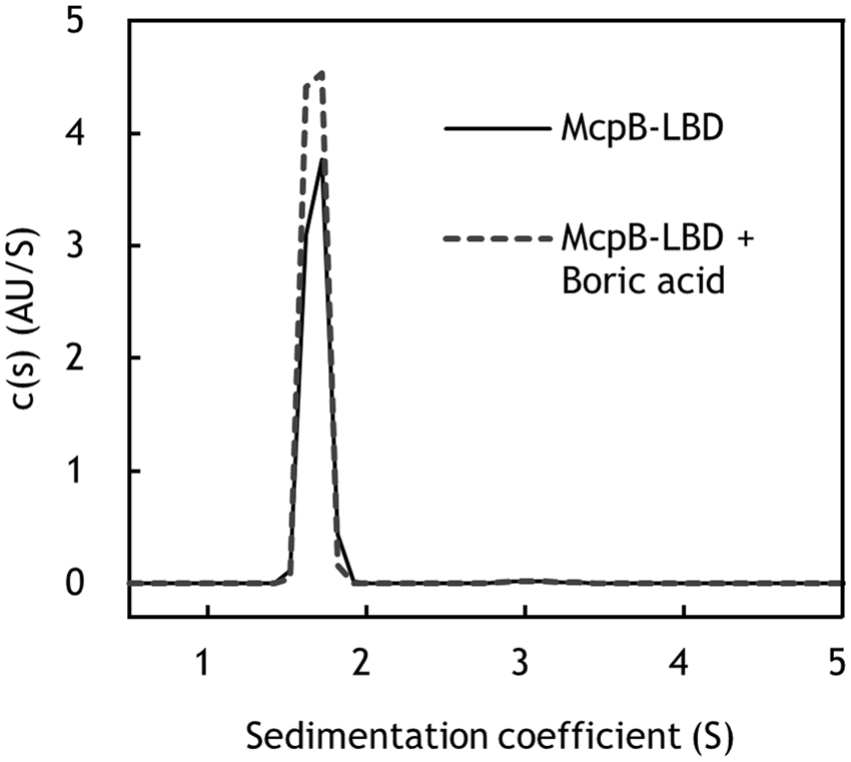
Determination of the oligomeric state of McpB LBD using analytical ultracentrifugation. Shown are sedimentation coefficient distributions for 1.5 mg/ml McpB LBD in the absence and presence of 1 mM boric acid.

As shown in Fig. 3, ITC analysis revealed that boric acid binds to the McpB LBD with an N value (binding ratio) of 0.4, suggesting that the McpB LBD dimer recognizes one boric acid molecule. This observation was confirmed by sedimentation velocity ultracentrifugation analysis. Figure 6 shows the sedimentation coefficient distribution obtained for the McpB LBD in the absence and presence of boric acid. In the absence of ligand, a single peak with an *s* value (standardized to 20 °C in water) of 2.53 S was observed, corresponding to an estimated molecular weight of 36.5 kDa, which is almost same size as an McpB LBD dimer. The sedimentation coefficient distribution of the McpB LBD in the presence of boric acid was essentially identical to that in the absence of boric acid. These results indicate that the McpB LBD is present exclusively as a dimer and recognizes one boric acid molecule.

### Biological significance of boric acid chemotaxis

The biological significance of chemotaxis in nature is generally that it enables bacteria to locate food sources. We therefore examined whether boric acid is essential for the growth of *R. pseudosolanacearum* Ps29. Strain Ps29 was cultured in plastic tubes with RSM medium containing different concentrations of boric acid (0–10 mM). There was no significant difference in growth at 0–1 mM boric acid, although growth at the highest concentration (10 mM) of boric acid was significantly higher (1.7 fold) than in the absence of boric acid (Fig. S3).

Chemotaxis is also important for virulence in *R. pseudosolanacearum* ^11, 12^. We investigated the role of McpB-mediated chemotaxis in tomato plant infection using a highly virulent strain of *R. pseudosolanacearum* (MAFF106611) and its *mcpB* deletion mutant (DMF11). Strain MAFF106611 also showed boric acid chemotaxis and DMF11 failed to respond to boric acid (Fig. S4A). A sand-soak inoculation experiment, in which cells of test strains are inoculated into sand away from the target plant, was conducted to assess plant infection by the *R. pseudosolanacearum* strains. We found that the *mcpB* deletion mutant of MAFF106611 was as infectious as wild-type MAFF106611 in this assay (Fig. S4B).

## Discussion

In this study, we conducted a detailed investigation of the positive chemotactic response of *R. pseudosolanacearum* Ps29 to “negative” control HEPES buffer and found that this response was directed toward boric acid leaching from borosilicate glass into the buffer. The most important finding of this study was the identification of boric acid as a novel chemoattractant. In addition, we identified the bacterial protein McpB as a chemosensor for boric acid by screening a library of *mcp* single-deletion mutants. The results of ITC assays examining the binding of boric acid to the LBD of McpB confirmed that this protein is a boric acid MCP.

MCP LBDs can be classified based on size as either cluster-I (120–210 aa) or cluster-II (220–290 aa) domains^29^. The putative LBD of McpB belongs to the cluster-I group, with a predicted LBD size of 157 aa and is annotated as 4-helix-bundle (4HB) in Pfam and InterPro. Protein structure predictions using the Phyre^2^ fold recognition server^30^ also suggested the presence of a 4HB domain in the *R. pseudosolanacearum* Ps29 McpB LBD (Fig. S1), similar to the structures predicted for McpM^12^ and McpT^23^, as well as *E. coli* Tar and Tsr^29^.

ITC analysis demonstrated that McpB LBD dimer binds one boric acid molecule. Although *P. aeruginosa* does not respond to boric acid, it exhibits chemotactic responses to phosphate, which has a similar chemical structure to boric acid^4^. We identified two CtpH and CtpL as MCPs for phosphate in *P. aeruginosa* ^31^. Rico-Jimènez *et al*. demonstrated that CtpL recognizes phosphate by biding of the periplasmic phosphate binding protein (PstS) in its phosphate loaded state, while CtpH binds directly phosphate^32^. They reported that CtpH LBD dimer bond one phosphate molecule. Since CtpH also has a 4HB in its LBD, binding of boric acid to McpB LBD has some parallels to binding of phosphate to CtpH LBD.

The binding of chemotaxis ligands to MCP LBDs initiates chemotactic signaling pathways that regulate the rotation direction of flagellar motors. The diversity of MCP ligand specificity reflects the diversity of LBD amino acid sequences. We performed a BLASTP analysis against the National Center for Biotechnology Information database using the McpB LBD as the query sequence. This similarity search indicated that several species of beta- and gamma-proteobacteria express proteins highly similar to the McpB LBD, most of which are putative MCPs. The *R. solanacearum* species complex, including strains GMI1000 (phylotype I), FQY_4 (phylotype I), SD54 (phylotype I), K60-1 (phylotype II), CFBP2957 (phylotype II), and PSI07 (phylotype IV), expresses McpB orthologues exhibiting a high degree of similarity to *R. pseudosolanacearum* Ps29 McpB LBD (>90% identity). Other beta-proteobacteria, such as *Parabukholderia* sp., *Massilia namucuonensis*, and *Burkholderia gladioli*, express MCPs with LBDs similar to that of McpB (approximately 50% identity). A number of gamma-proteobacteria express proteins with McpB LBD homologous sequences (up to 68% identity), including *Dickeya* sp., *Cedecea neteri*, *Pectobacterium carotovorum*, *Erwinia* sp., *Xanthomonas* sp., and *Pseudomonas syringae*. Interestingly, most of these bacteria are plant pathogens.

Boron serves as a micronutrient in prokaryotes and eukaryotes. Some bacteria produce biologically active compounds containing boron^33^. For example, *Streptomyces antibioticus*, *Streptomyces griseus*, and *Sorangium cellulosum* produce boromycin^34^, aplasmomycin^35^, and tartrolons^36^, respectively, all of which are antibiotics active against gram-positive bacteria. Many gram-positive and -negative bacteria produce furanosyl borate diester (known as autoinducer-2), which functions as a signaling compound in cell-to-cell communication^37^. Boron is also involved in the growth of nitrogen-fixing bacteria. Heterocystous cyanobacteria (*Nodularia* sp., *Chlorogloeopsis* sp., and *Nostoc* sp.) and actinomycetes *Frankia* strain BCU110501 require boron for growth under nitrogen-fixing conditions^38, 39^. Boron plays a role in the stabilization of heterocysts in cyanobacteria and vehicle envelopes in *Frankia*, which are essential for the exclusion of nitrogenase-poisoning oxygen. Boron is reportedly required for the establishment of effective legume-*Rhizobium* symbiosis. In addition, boron is necessary for maintaining the cell wall structure of nodules^40^ and the development of infection threads and nodule invasion^41^. In higher plants, boron is an essential micronutrient, as it is required for maintaining cell wall integrity^42^. The major components of plant cell walls are cellulose, hemicellulose, and pectin polysaccharides. Borate cross-links two chains of the pectin polysaccharide rhamnogalacturonan II by binding to their apidose residues; this cross-linking contributes to the maintenance of cell wall integrity^43^.

What biological significance does boric acid chemotaxis have? Chemotaxis toward boric acid could be a “fortuitous” response mediated by McpB. In a previous study, we identified McpT as an MCP for L-tartrate, which *R. pseudosolanacearum* Ps29 can utilize as a sole carbon source^23^. McpT recognizes D-malate, an unnatural enantiomer of malate, as a strong attractant, although strain Ps29 cannot utilize this compound. We concluded that chemotaxis toward D-malate is a fortuitous response associated with McpT. To more clearly determine whether boric acid chemotaxis is a fortuitous response, we analyzed the chemotaxis of wild type strain Ps29 to several compounds with similar structures to boric acid. Any of these compounds did not attract cells, suggesting that boric acid chemotaxis is not a fortuitous response.

Many attractants are growth substrates, for example amino acids, organic acids, sugars, and phosphate. Boric acid could be an important nutrient for the growth of *R. pseudosolanacearum* Ps29. We therefore confirmed whether there were differences in growth in a defined medium containing different concentration of boric acid. At 10 mM boric acid, cell growth was finally improved although it was inhibited at an early stage of growth (Fig. S3). However, because 10 mM is not an environmentally relevant concentration with respect to boron (5 mg/kg in basalts; 100 mg/kg in shales^44^), the observed enhanced growth in 10 mM boric acid is probably not environmentally significant.

Chemotaxis also plays an important role in facilitating ecological interactions, including plant infection by *R. solanacearum* ^11, 12^. In a previous study, we demonstrated that chemotaxis toward L-malate enables *R. pseudosolanacearum* to locate and interact with tomato roots and is required for subsequent infection of tomato plants^12^. The distribution of McpB orthologues is limited to plant pathogenic bacteria belonging to the beta- and gamma-proteobacteria, and borate is a ubiquitous constituent of higher plants. These data suggest that boric acid functions as a chemotactic signaling compound that facilitates interactions between bacterial cells and plants. To confirm this possibility, we conducted infection assay using sand-soak inoculation method. In this assay, the bacteria must traverse a distance and locate and invade the host plant in order to infect plants because cell suspension was inoculated into the sand at a spot ~30 mm away from tomato seedling. The infectivity of strain DMF11 (MAFF106611 *mcpB* deletion mutant) did not differ significantly from that of wild-type MAFF106611 (Fig. S4B). However, this result does not rule out the possibility that McpB-mediated chemotaxis is involved in plant infection, because borate is included in Plant Nutrient Solution (PNS)^45^ used to support the growth of tomato plants. Therefore, a novel assay system should be developed to assess the role of boric acid chemotaxis in the infection of plants by *R. pseudosolanacearum*.

In this study, we found *R. pseudosolanacearum* Ps29 was attracted to boric acid and identified McpB as a chemotaxis sensor for boric acid. This is the first report of a biological boric acid sensor. We demonstrated that boric acid directly bound to LBD of McpB.

## Materials and Methods

### Bacterial strains, plasmids, and culture conditions

The bacterial strains and plasmids used in this study are listed in Table S1. Highly motile *R. pseudosolanacearum* Ps29 and its derivatives were used for chemotaxis research, and *R. pseudosolanacearum* MAFF106611 and its derivatives were used for tomato plant infection assay. *E. coli* JM109 and BL21(DE3) were used for plasmid construction and protein expression, respectively. The *R. pseudosolanacearum* Ps29 strains were grown in *R. solanacearum* minimal (RSM) medium^12^ after preculture in CPG medium^46^ at 28 °C. *P. aeruginosa* PAO1 was cultured in T0 medium^31^ after preculture in Luria-Bertani (LB) medium at 37 °C. *E. coli* strains were cultured in LB medium. When necessary, 40 μg/ml of kanamycin was added.

### Chemotaxis assay

Computer-assisted capillary assays were carried out as described previously^24^. Cell movement was observed under an inverted microscope. Cells in a 10-μl suspension were placed on a coverslip, and the assay was initiated by placing the coverslip upside down on a U-shaped spacer to fill the chemotaxis chamber in the presence of a glass capillary containing test compound plus 1% (wt/vol) agarose. Cells were videotaped, and the number of bacteria migrating toward the mouth of the capillary at the initial time (N_0_) and at each given time interval (N_t_) was determined using digital image processing. The strength of the chemotactic response was determined and reported in terms of normalized cell number per frame (N_t_/N_0_). The chemotaxis buffer was 10 mM HEPES (pH 7.0, unless stated otherwise).

### Complementation of *mcpB*

To construct the pPS11 plasmid for use in the complementation analysis, a 1.9-kb region encoding the RS_RS17100 (old locus tag RSc3412) homolog gene (*mcpB*) of *R. pseudosolanacearum* Ps29 was amplified by PCR using the CLRS11f/CLRS11r primer pair (Table S2). The amplified fragments were digested with *Eco*RI + *Bam*HI and cloned between the *Eco*RI and *Bam*HI sites of pRCII^12^. pPS11 was then introduced into strain DPS11 by electroporation, as described previously^12^.

### Expression and purification of the McpB-LBD

A DNA fragment encoding the McpB-LBD (aa 30-186) was amplified using the McpB_LBDf/McpB_LBDr primer pair (Table S2). The amplified fragments were digested with *Nde*I + *Bam*HI and cloned between the *Nde*I and *Bam*HI sites of pET28b(+) (Novagen) to construct pET28_PsMcpB_LBD, which was then used to transform *E. coli* BL21(DE3). The transformed strain was cultured in LB medium supplemented with 40 μg/ml of kanamycin at 28 °C. After reaching an OD_600_ value of 0.5, 0.1 mM IPTG was added to induce expression of the McpB LBD, and cultivation was continued overnight at 18 °C. The bacteria were harvested by centrifugation and lysed using B-PER^TM^ Bacterial Protein Extraction Reagent (Thermo Fisher Scientific). After centrifugation at 20,000 × *g* for 1 h, the supernatant was loaded onto a His GraviTrap TALON column (GE Healthcare) equilibrated with buffer (20 mM sodium phosphate, 500 mM NaCl, pH 7.0) containing 30 mM imidazole. The protein was eluted with the same buffer containing 300 mM imidazole.

### ITC

For ITC, Vivaspin 20 (10 kDa molecular weight cutoff, GE Healthcare) were used to exchange purified McpB LBD into ITC buffer (20 mM sodium phosphate, 100 mM NaCl, 10% (v/v) glycerol, pH 7.0). ITC experiments were performed on a MicroCal iTC_200_ isothermal titration calorimeter (GE Healthcare) at 25 °C. Test compounds were dissolved in ITC buffer. Protein solution (200 μM) was added to fill the sample cell and titrated with 1 mM compound solution. Data were analyzed using the One Set of Sites model of the MicroCal version of ORIGIN 7.0 software.

### CD analysis

For CD analyses, Vivaspin 20 columns (10 kDa molecular weight cutoff, GE Healthcare) were used to exchange purified McpB LBD into CD buffer (20 mM sodium phosphate, 100 mM NaCl, pH 7.0). CD experiments were performed on a Jasco J-820 CD spectrometer (Tokyo, Japan) equipped with a 1-mm path length cuvette using 20 μM McpB LBD in the absence and presence of 100 μM boric acid. CD spectra (190–260 nm) were recorded at 25 °C. For thermal denaturation experiments, CD at 222 nm was monitored from 20 to 70 °C.

### Analytical ultracentrifugation

For analytical ultracentrifugation analyses, Vivaspin 20 (10 kDa molecular weight cutoff, GE Healthcare) were used to exchange purified McpB LBD into ITC buffer. Sedimentation velocity experiments were performed using an Optima XL-I analytical ultracentrifuge (Beckman Coulter, Brea, CA, USA) with a 4-chamber An60Ti rotor at 42,000 rpm at 25 °C. Concentration gradients were measured by UV absorption at 280 nm without a time interval between successive scans. The data were analyzed using SEDFIT^47^.

### Plant infection assay

Virulence of *R. pseudosolanacearum* MAFF106611 strains were tested using the sand-soak inoculation method^12^. Sterile tomato (Solanum lycopersicum cv. Oogata-fukuju) seeds were kept overnight at 4 °C in the dark in order to synchronize germination. Seeds then were placed onto petri dishes containing PNS solidified with 1.5% (w/v) agar and allowed to grow in a climate-controlled growth chamber (Sanyo) for 7 days at 28 °C with a 16:8 h light:dark cycle. Bacterial cells frown in RSM medium for 20 h were collected (3,300 × *g* for 2 min), washed twice with sterile deionized water and adjusted to a final density of approximately 10^6^ CFU/ml. Seven-day-old tomato roots were wounded by cutting 1 cm away from the base of the stem. The wounded seedling was planted at the center of plant box (6 × 6 × 10 mm) containing 140 g quarts sand and 35 ml PNS, while 50 μl of cell suspension was inoculated near wall of the plant box. The plants were maintained in a climate-controlled growth chamber at 28 °C with a 16:8 h light:dark cycle for 10 days and observed daily. All virulence assays included 8 plants per treatment, and each experiment was repeated at least eight times.

## Electronic supplementary material

Supplementary Information
