# Supplementary material for: Identification of boric acid as a novel chemoattractant and elucidation of its chemoreceptor in Ralstonia pseudosolanacearum Ps29
Source: Sci Rep. 2017 Aug 17;7:8609. doi: 10.1038/s41598-017-09176-3 (PMC5561266; doi:10.1038/s41598-017-09176-3)
Supplement: Supplementary file 1 — Supplementary Information [file 41598_2017_9176_MOESM1_ESM.pdf]

Identification of boric acid as a novel chemoattractant and elucidation of its chemoreceptor in *Ralstonia pseudosolanacearum* Ps29

Akiko Hida, Shota Oku, Yutaka Nakashimada, Takahisa Tajima & Junichi Kato

Department of Molecular Biotechnology, Graduate School of Advanced Sciences of Matter, Hiroshima University, Higashi-Hiroshima, Hiroshima 739-8530, Japan

Corresponding author:

E-mail: jun@hiroshima-u.ac.jp

Tel: +81824247757

Supplementary Information

Supplemental material:

Table S1 Bacterial strains and plasmids used in this study.

Strain and plasmid	Relevant characteristic(s)[a]	Reference
Bacterial strains		
R. pseudosolanacearum		
Ps29	Wild type strain race 1, biovar 3, phylotype I	[1]
DPS11	Ps29 derivative; Δ*mcpB*	[2]
MAFF106611	Wild type strain race 1, biovar 4, phylotype I	[1]
DMF11	MAFF106611 derivative; Δ*mcpB*	This study
P. aeruginosa		
PAO1	Wild-type strain	[3]
E. coli		
JM109	*recA1*, *endA1*, *gyrA96*, *thi-1*, *hsdR17* ($r_k^- m_k^+$), *e14*$^-$ (*mcrA*$^-$), *supE44*, *relA1*, Δ(*lac-proAB*)/F’ [*traD36*, *proAB*$^+$, *lacI*^{q}, *lacZ* ΔM15]	[4]
BL21(DE3)	F$^-$, *ompI*, *hsdS*$_B$ (r_B^- m_B^-)	[5]
Plasmids		
pRCII	*E. coli-Ralstonia* shuttle vector derived from pKZ27; IncQ, *lac* promoter; Km^r	[2]
pPS11	pRCII with a 1.9-kb PCR fragment including *mcpB* of Ps29	This study
pET28b(+)	Km^r, protein expression vector	Novagen
pET28_PsMcpB_LBD	pET28b(+) with a 471-bp PCR fragment encoding McpB LBD of Ps29	This study

[a] Km^r, kanamycin resistance

Table S2 Oligonucleotides used in this study.

Oligonucleotide	Sequence (5’-3’)
CLRS11f	ATGAATTCTAGCGCGTCACTCAAGAAAGG
CLRS11r	ATGGATCCAAGACATGGAAGCCAAGCTG
McpB_LBDf	AATTCATATGGGGCGCCAGGCCGCCGCGAC
McpB_LBDr	AATTGGATCCTTAGCGGGCCAATGCTGCCG

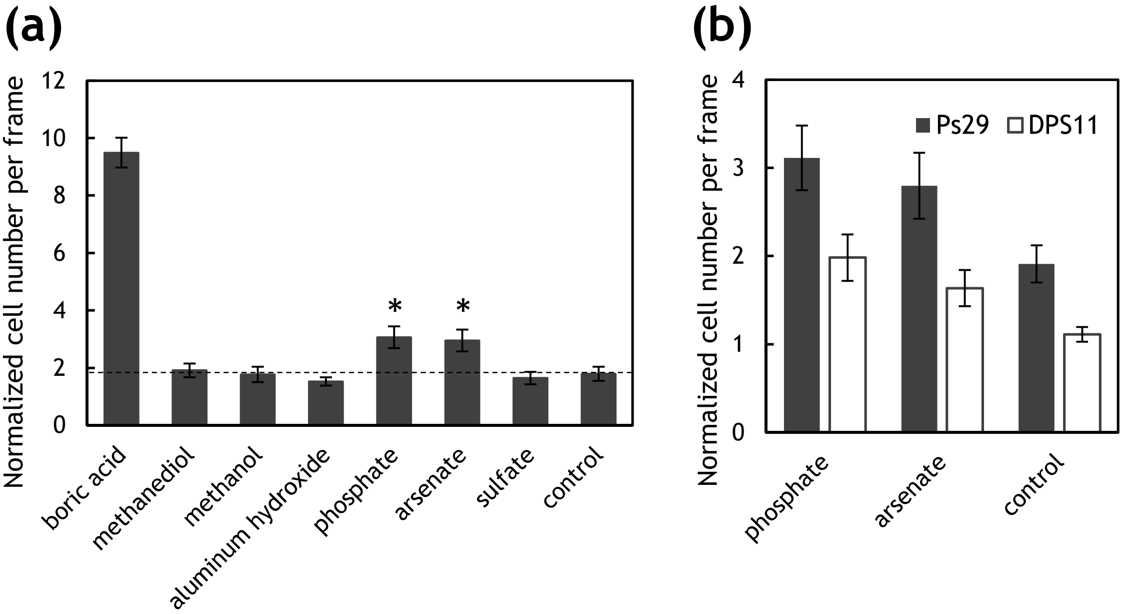

Fig. S1 Chemotaxis by *R. pseudosolanacearum* Ps29 strains to compounds with similar structure to boric acid. **(a)**, responses by wild type strain. **(b)**, responses to phosphate and arsenate by wild-type and *mcpB* deletion mutant (DPS11). Boric acid, 0.5 mM; other compounds other than aluminum hydroxide, 5mM; aluminum hydroxide, supernatant of suspension (because of low solubility). Control indicates 10 mM HEPES buffer. The normalized cell number was calculated by dividing the number of bacterial cells observed at 1 min by the number observed at the initiation of the experiment. Vertical bars represent the standard error of measurement for experiments performed at least in triplicate. Asterisks indicate significant difference compared with the responses to control ($P < 0.05$ by Student's t-test).

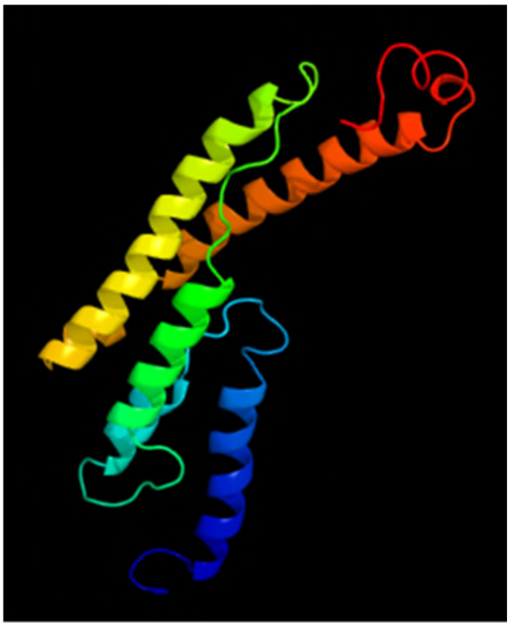

Fig. S2 The predicted three-dimensional (3D) structure of the *R. pseudosolanacearum* Ps29 McpB LBD. The 3D structure was predicted using the Phyre^2 algorithm (intensive mode). Blue, N-terminus; red, C-terminus.

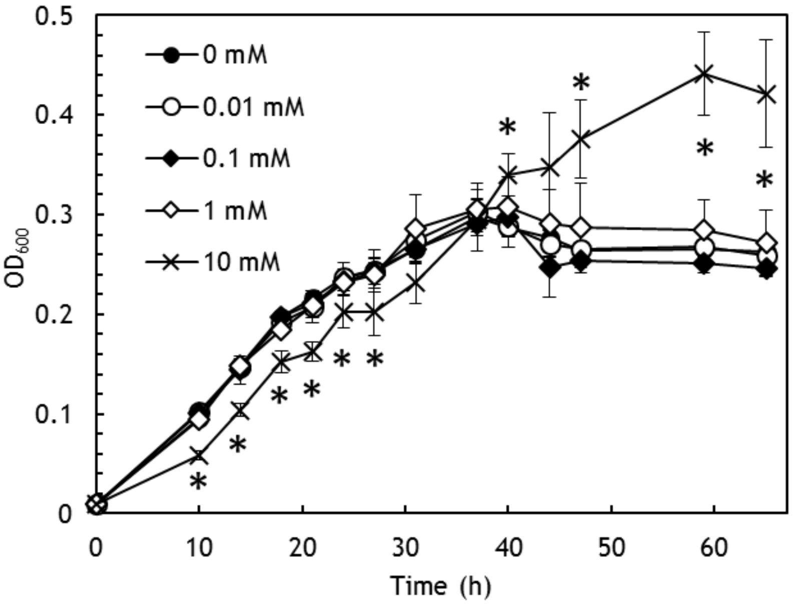

Fig. S3 Growth curves for wild type *R. pseudosolanacearum* Ps29 in RSM medium containing 5 g/l glucose and 0 to 10 mM boric acid. Vertical bars represent the standard error of measurement for triplicate experiments. Asterisks indicate significant differences in growth of the wild-type strain in the absence of boric acid and in the presence of 10 mM boric acid (Student's *t*-test, $P < 0.05$).

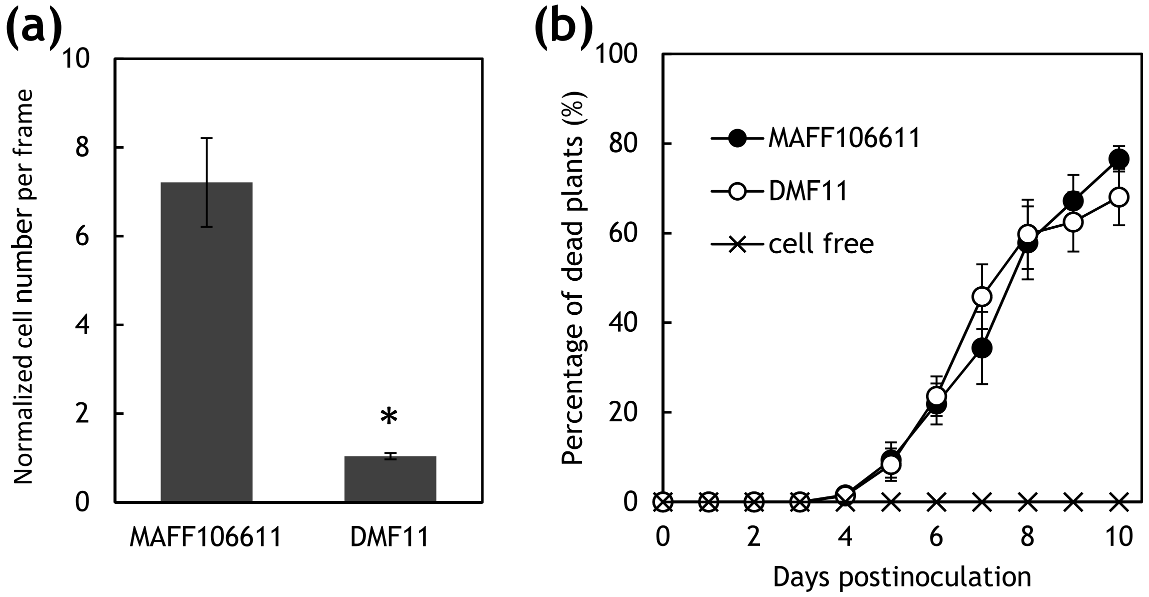

Fig. S4 Analyses using *R. pseudosolanacearum* MAFF106611 wild-type and *mcpB* deletion mutant (DMF11). **(a)**, chemotactic responses to 5 mM boric acid. The normalized cell number was calculated by dividing the number of bacterial cells observed at 2 min by the number observed at the initiation of the experiment. Asterisk indicates significant difference compared with the response by wild-type (P < 0.05 by Student's t-test). **(b)**, Sand-soak inoculation virulence assay. There was no significant difference in the percentage of dead plants.

Reference

1. Yamada, T. *et al*. New bacteriophages that infect the phytopathogen *Ralstonia solanacearum*. *Microbiology* **153**, 2630-2639 (2007).

2. Hida, A. *et al*. Identification of the *mcpA* and *mcpM* genes encoding methyl-accepting proteins involved in amino acid and L-malate chemotaxis, *Ralstonia pseudosolanacearum* (Formerly *Ralstonia solanacearum* Phylotypes I and III). *Appl. Environ. Microbiol.* **81**, 7420-7430 (2015).

3 Holloway, B.W., Krishnapillai, V. & Morgan, A.F. Chromosomal genetics of *Pseudomonas*. *Microbiol. Rev.* **43**, 73-102 (1979).

4. Sambrook, J., Fritsch, E.F. & Maniatis, T. Molecular cloning a laboratory manual, 2nd ed. (Cold Spring Habor Laboratory Press, Cold Spring Harbor, NY, USA., 1989)

5. Studier, F.W. & Moffatt, B.A. Use of bacteriophage T7 RNA polymerase to direct selective high-level expression of cloned genes. *J. Mol. Biol.* **189**, 113-130 (1986).
